# Non-thermal plasma causes *Pseudomonas aeruginosa* biofilm release to planktonic form and inhibits production of Las-B elastase, protease and pyocyanin

**DOI:** 10.3389/fcimb.2022.993029

**Published:** 2022-09-23

**Authors:** Petra Kašparová, Eva Vaňková, Martina Paldrychová, Alžběta Svobodová, Romana Hadravová, Irena Jarošová Kolouchová, Jan Masák, Vladimir Scholtz

**Affiliations:** ^1^ Laboratory of Applied Biology, Department of Biotechnology, University of Chemistry and Technology in Prague, Prague, Czechia; ^2^ Laboratory of Non-thermal Plasma, Department of Physics and Measurements, University of Chemistry and Technology in Prague, Prague, Czechia; ^3^ Viral and Microbial Proteins, Institute of Organic Chemistry and Biochemistry of the Czech Academy of Sciences, Prague, Czechia

**Keywords:** antivirulence factors, biofilm disruption, cold atmospheric plasma (CAP), combined therapy, haemolytic activity

## Abstract

The increasing risk of antibiotic failure in the treatment of *Pseudomonas aeruginosa* infections is largely related to the production of a wide range of virulence factors. The use of non-thermal plasma (NTP) is a promising alternative to antimicrobial treatment. Nevertheless, there is still a lack of knowledge about the effects of NTP on the virulence factors production. We evaluated the ability of four NTP-affected *P. aeruginosa* strains to re-form biofilm and produce Las-B elastase, proteases, lipases, haemolysins, gelatinase or pyocyanin. Highly strains-dependent inhibitory activity of NTP against extracellular virulence factors production was observed. Las-B elastase activity was reduced up to 82% after 15-min NTP treatment, protease activity and pyocyanin production by biofilm cells was completely inhibited after 60 min, in contrast to lipases and gelatinase production, which remained unchanged. However, for all strains tested, a notable reduction in biofilm re-development ability was depicted using spinning disc confocal microscopy. In addition, NTP exposure of mature biofilms caused disruption of biofilm cells and their dispersion into the environment, as shown by transmission electron microscopy. This appears to be a key step that could help overcome the high resistance of *P. aeruginosa* and its eventual elimination, for example in combination with antibiotics still highly effective against planktonic cells.

## Graphical Abstract

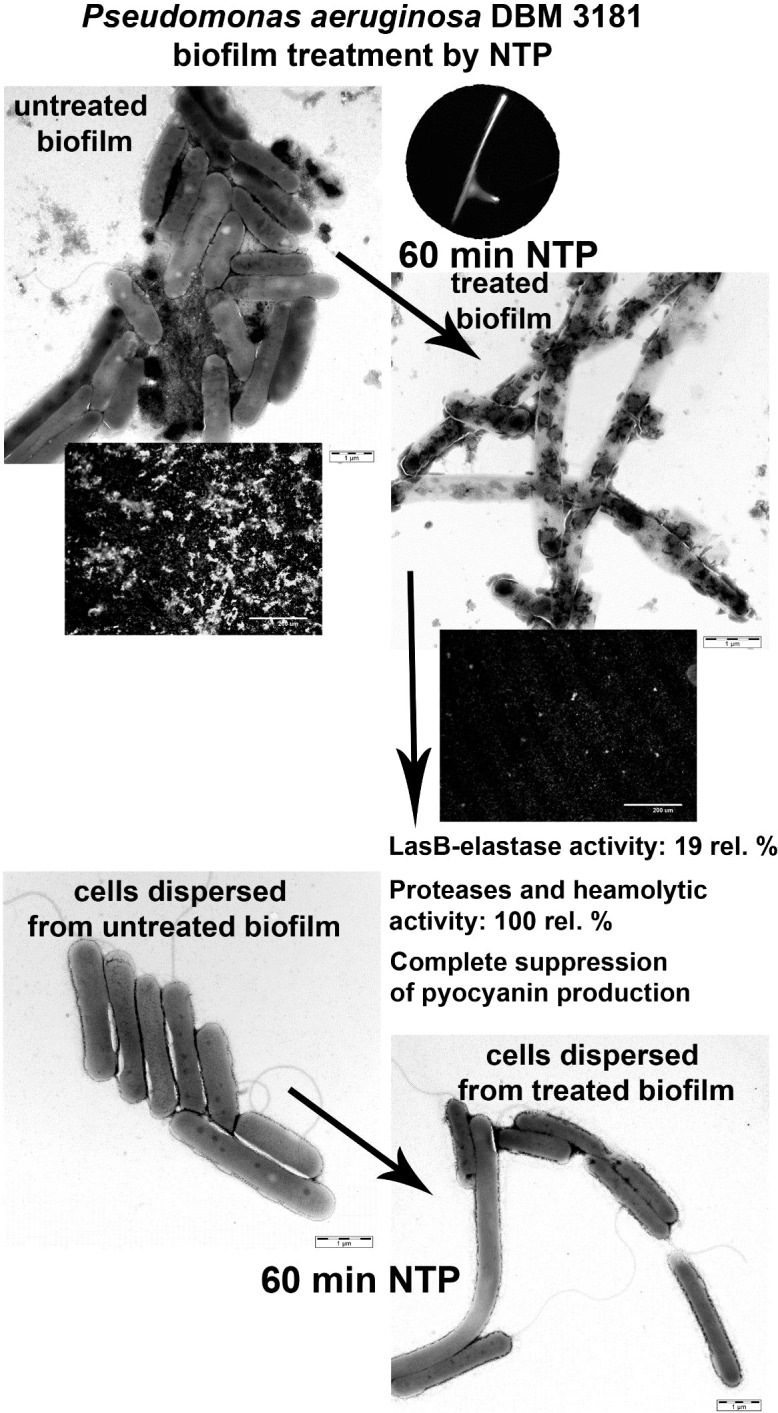

## Introduction

*Pseudomonas aeruginosa* belongs to the most infamous pathogens in presence included in the group of highly virulent and antibiotic resistant bacterial pathogens with acronym ESKAPE (*Enterococcus faecium*, *Staphylococcus aureus*, *Klebsiella pneumoniae*, *Acinetobacter baumannii*, *Pseudomonas aeruginosa*, and *Enterobacter* spp.) (Rice, 2008). It is responsible for a wide spectrum of both acute and chronic infectious diseases occurring in human body. There are several comprehensive reviews dealing with its pathogenesis and clinical impact (Rice, 2008; Christophersen et al., 2012; Gellatly and Hancock, 2013; Behzadi et al., 2021). In short, the pathogen can cause various respiratory tract infections (pneumonia, chronic obstruction lung disease, and cystic fibrosis related infections), infections of soft tissues (diabetic ulcer or open wound infections), urinary tract infections due to catheterization, ear infections (otitis externa et media), keratitis or systemic bacteraemia in immunocompromised patients. Many of the infectious diseases have nosocomial manner and often relate to biofilm formation of the species (Gellatly and Hancock, 2013). Current options for treatment of mentioned pathological states are very limited due to both intrinsic and adapted antibiotic resistance of *P. aeruginosa* to many antibiotic classes (β-lactams and cationic antimicrobial peptides or aminoglycosides and polyketides like tetracyclines or macrolides) (Scholtz et al., 2021). Lately, only fluoroquinolones, third and fourth generations of β-lactams are used. Also polymyxins like colistin (polymyxin E) and polymyxin B have been once again introduced to clinical practice even though they were previously limited due to their nephrotoxicity (Zhao et al., 2020). To prevent the emergence of adapted resistance, a combined drug therapy has been used, nevertheless the resistance eventually developed also (Kingsbury et al., 2012; Mu et al., 2014; Bandeira et al., 2018; Vaňková et al., 2020a). The new probably strikingly different treatment approach is sought because the drug development of novel antibiotic classes is very slow in contrast to antibiotic resistance spread.

High pathogenicity of *P. aeruginosa* is entailed to a wide array of virulence factors which facilitates the infectivity of the species and helps the cells proliferate in the host tissues while evading the immune system response (Balasubramanian et al., 2013; Behzadi et al., 2021). A major virulence factor contributing to significant persistence of *P. aeruginosa* infections is its ability to adhere to both biotic and abiotic surface and form a thick often mucous biofilm (Tre-Hardy et al., 2008; Bjarnsholt et al., 2009; Ma et al., 2009; Das et al., 2016; Kim et al., 2018). The cell surface adhesion is enabled by outer membrane organelles such as a single monopolar flagella, type IV pili, fimbriae (all responsible for a swarming and a twitching motility of the species) and surface adhesins (Gellatly and Hancock, 2013). The cells of *P. aeruginosa* adhere especially to either hydrophobic or porous materials making for example joint implants or porous lung epithelia very suitable for the adhesion. In later stages of biofilm proliferation, quorum sensing (QS) further regulates the virulence manifestation as the high cell density environment enables a cell-to-cell communication *via* autoinducers (Juhas et al., 2005; Lee and Zhang, 2015). *P. aeruginosa* possesses four distinct QS systems with strict hierarchy with the *las* system at the top, followed by *rhl* system (both acting *via* acyl-homoserine lactones), *pqs* or *iqs* system. QS is responsible for further virulence manifestation of the species and regulates production of extracellular virulence factors like proteases, lipases, phospholipases, haemolysins and other cytotoxins. *Las* system controls production of LasA elastase (serine protease often termed staphylolysin) and LasB-elastase (often termed just elastase, prevents phagocytosis in host lungs) (Defoirdt, 2018; Haque et al., 2018). *P. aeruginosa* produced also alkaline protease responsible for degradation of free flagellin molecules (switch of motile cells to sessile form of growth during biofilm formation thus limiting the detection by host immune system). Lastly, also protease IV is produced, which is a serine protease responsible for degradation of complement proteins, immunoglobins and fibrinogen also helping immune system evasion (Gellatly and Hancock, 2013). Among other virulence factors, lipases and haemolytic phospholipases contribute to overall virulence and infectivity of the species *via* breaking surfactant lipids in the membrane of host cells thus causing the disruption of their membranes (Behzadi et al., 2021). In addition, these hydrolases are usually responsible also for the dispersion of cells from the mature biofilms. Such dispersion is the last and final stage of biofilm formation usually mediated by limited nutrients, high shear forces, large cell density or antimicrobial treatment. The cells dispersed from the biofilm occurs in planktonic form and represent a very metabolically active form of growth that can further disseminate to other niches, tissues throughout the host’s body and colonize new areas. Such spread significantly enhances the possibility systemic bacteraemia development and improve the persistence of pathogen’s presence in the body. Nevertheless, as pointed out above, planktonic cells are also significantly more susceptible towards any antiseptic/antimicrobial treatment and thus dispersion of cells from biofilm might be a suitable strategy for increment of therapeutical efficiency (Juhas et al., 2005; Bjarnsholt et al., 2009). *P. aeruginosa* produces also colourful pigments like pyocyanin or pyoverdine. Pyocyanin causes oxidative stress towards host cells by disruption of host catalase and mitochondrial electron transport (Wang et al., 2020). Pyoverdine is a siderophore responsible for iron chelation helping the chronic infection development in host. Taken together, *P. aeruginosa* has plenty of weapons and other means for development of infections in host and further proliferation to chronic state. It is not surprising that novel drug development focusses on antivirulence therapy of infectious diseases in general. The antivirulence therapy either suppresses the ability to cause infection in the first place or the option to harm host even more. Most importantly, antivirulence drugs do not specifically kill the pathogen’s cells but only attenuate their pathogenicity (Defoirdt, 2018; Khan et al., 2019). Thus, they do not put a great selective pressure on the treated population as in the case of common antibiotics and the development of resistance is greatly reduced. The current antivirulence drug development is strongly focused on the regulatory level of virulence and the agents with possible anti-QS effect are very lucrative (Khan et al., 2019).

Non-thermal plasma (NTP) treatment emerged as an elegant agent with possible antivirulence action as it is known to suppress biofilm formation of a wide range of microorganisms (Julák et al., 2018). Plasma is a fourth state of matter consisting of ionized particles. NTP is a specific type of plasma, which energy is primarily stored into the free electrons while the temperature of neutrals and ionized particles remains low. For biological application, NTP is typically generated by electric discharges at ambient conditions (air atmosphere, atmospheric pressure, temperature of 40 °C at maximum) (Julák et al., 2012; Vaňková et al., 2019). The free electrons accelerated by electric field interact with the gas particles producing reactive gas species, which are quite toxic towards treated bacterial or fungal cells creating great oxidative stress in the process. NTP is readily used for both decontamination of medical tools like joint implants, surgical utensils and catheters before use or for the treatment of open wounds, dermatitis, biofilm-related infections etc. (Moreau et al., 2008; Julák et al., 2018; Lopez et al., 2019). It was also recommended for decontamination of food packaging or abiotic materials (e.g., protective respiratory masks) (Scholtz et al., 2015). Its antimicrobial activity and possible applications were comprehensively described in several reviews (Liao et al., 2017; Gupta and Ayan, 2019; Scholtz et al., 2021). We have previously verified great antibiofilm effect of NTP towards various species, especially *P. aeruginosa* (Paldrychová et al., 2019; Vaňková et al., 2019; Julák et al., 2020; Paldrychová et al., 2020), and in continuation, the presented study observed whether NTP would affect virulence manifestation of *P. aeruginosa* (ability to re-form biofilm, produce Las-B elastase, proteases, haemolysins, lipases, gelatinase or pyocyanin) or its QS system. This study could contribute to the further spread of NTP as an efficient antimicrobial, antivirulence or anti-QS tool for the treatment of infectious diseases caused by *P. aeruginosa*.

## Materials and methods

### Microorganisms

*P. aeruginosa* DBM 3081 (CCM 1968, soil isolate) and *P. aeruginosa* DBM 3181 (a clinical isolate from Military University Hospital, Prague, Czech Republic) were kindly provided by the Department of Biochemistry and Microbiology of University of Chemistry and Technology in Prague, Czech Republic. The strains *P. aeruginosa* ATCC 10145 (type strain, very mucous and pyocyanin positive) and *P. aeruginosa* ATCC 15442 (strain used for antimicrobial testing and food control), were acquired from Czech National Collection of Type Cultures (CNCTC). The differences between strains are vital for better assessment of virulence factor production and how it is affected by NTP treatment. The initial inoculum of each strain was cultured in Luria Bertani (LB) Broth for 24 h at 37 °C and 150 rpm.

### Non-thermal plasma generation

A device producing a DC cometary discharge was used to generate NTP, as described previously (Scholtz et al., 2013; Julák et al., 2020). Briefly, this device was composed of two needle electrodes connected to 5 kV power supply (UNI-T UT 513A, UNI-TREND, China) in a mutual position of approximately 30°, 5 mm apart with insulated metallic grid between the discharge and exposed object. The distance between electrodes and object and between the grid and object was approximately 30 and 10 mm, respectively. The device was operated at 50-70 μA, 5 kV in open air at laboratory temperature.

### Biofilm formation

Biofilm was prepared on Ti-6Al-4V alloy coupons according to Vaňková et al. (2019). Briefly, cultured inoculum was adjusted to OD_600nm_ = (0.600 ± 0.020) which corresponds to approximately 3×10^7^ CFU/ml. Adjusted cell suspension in a volume of 3 ml was poured into a sterile plastic container (7×3 cm; volume 40 ml) by P-LAB (Czech Republic) containing a sterile Ti-6Al-4V alloy coupon (15 mm in diameter) by Prospon (Czech Republic). The alloy is routinely used material for medical implant manufacturing. The coupons were sterilized by sonication in EDTA : SDS (1:1) solution for 30 min, thoroughly rinsed with distilled water, sterilized in an autoclave at 121 °C for 20 min and then let desiccate in a dryer (105 °C) overnight before experiment. No nourishments or other treatments were applied to the coupons before culture. The biofilms on Ti-6Al-4V coupons were formed for 24 h at 37 °C and 150 rpm. After cultivation, the biofilm-covered coupons were removed from containers, rinsed with saline, and put to sterile petri dish for NTP treatment.

### Susceptibility of biofilm to NTP treatment

The rinsed biofilms of *P. aeruginosa* on Ti-6Al-4V coupons were put into petri dish as described above. The samples were treated with NTP for 15, 30 and 60 min on both sides (30, 60 and 120 min in total). To verify the ability of the biofilm to survive NTP exposure, each treated sample was put in sterile plastic container with new portion of growth medium and the cultivation continued for another 24 h at 37 °C and 150 rpm. In addition to determining the viability of biofilm cells, an important factor in terms of the biofilm’s survival ability is also the verification of the viability of the cells dispersed from the biofilm. Therefore, at the end of the cultivation, these cells were taken from the vicinity of the biofilm and subjected to determination of their viability using several methods described in the next chapters.

### Viability of biofilm cells affected by NTP

To evaluate the viability of biofilm cells, the NTP-treated biofilm on Ti-6Al-4V coupons was cultivated for another 24 h after NTP exposure and then thoroughly rinsed with saline before each method used.

The survival of biofilm cells was evaluated by colony forming units per ml (CFU/ml) counting. In short, each coupon was put into glass container (4.5×2.5 cm; volume 22 ml) with 1 ml of saline and sonicated for 15 min to release the biofilm cells from the coupon surface. The biofilm cells in the saline were subdued to decimal dilution and poured on LB agar plates. CFU/ml were counted as described in detail in Vaňková et al. (2020b). The inoculated agars were incubated for 24 h at 37 °C.

The metabolic activity of biofilm cells was evaluated by viability assay previously described in detail in Julák et al. (2020). Briefly, coupon with rinsed biofilm cells was put into glass container with 500 μl of filtered solution of 3-(4,5-dimethyl-1,3-thiazol-2-yl)-2,5difenyltetrazolium bromide (MTT, 1 mg/ml in PBS; Across Organics, USA) and 600 μl of D-Glucose (57.4 mg/ml in PBS; Penta, Czech Republic). The cells were incubated at 37 °C and 150 rpm for 2 h. A formazan dissolving solution (160 g/l SDS (Carl Roth, Germany), 40% (v/v) DMF (Penta, Czech Republic) in 2% (v/v) acetic acid (Penta, Czech Republic) was poured to mixture in a volume of 1,000 μl and the incubation continued at 37 °C and 230 rpm for approximately 30 min until complete solubilization of formazan crystals. A 100 µl aliquot of the dissolved formazan was spectrophotometrically measured at 570 nm. The experiments were carried out in three independent repetitions.

### Visualization of biofilm cells affected by NTP using spinning disc confocal microscopy

The effect of NTP on viability of *P. aeruginosa* biofilm cells was visualized with LIVE/DEAD kit by spinning discs confocal microscopy (SDCM). Fluorescence probes SYTO13 (Invitrogen, USA), and propidium iodide (PI; Sigma-Aldrich, USA) were used for the biofilm staining according to manufacturer’s recommendations. The biofilm was cultured on Ti-6Al-4V coupons, treated with NTP as previously described and rinsed with saline before staining with selected probes in the dark for 10 min. The excess dye was rinsed away, and the samples were placed onto petri dish suitable for microscopy (MatTek, USA) with the stained side down and visualized by the inverse microscope Olympus/Andor (Japan/Ireland). Final figures are depicted with scale bar 200 µm.

### Viability of cells dispersed from biofilm affected by NTP

To examine the complete effect of NTP treatment on the ability of biofilm to survive its action, the viability of cells dispersed from the biofilm to a new portion of the growth medium after another 24 h of cultivation was determined. This procedure enables to assess whether the cells dispersed from the biofilm affected by NTP can regenerate and possibly start a new biofilm formation cycle. Therefore, the surrounding suspension of cells above treated biofilm was harvested at the end of the cultivation and subjected to further analyses.

The turbidity of resulting suspension was measured spectrophotometrically at 600 nm (Infinite M200 Pro Reader, Tecan, Switzerland).

The survival of cells dispersed from the biofilm was evaluated by CFU/ml counting. A volume of 1 ml of cells’ suspension surrounding Ti-6Al-4V coupons covered by biofilm in plastic container at the end of the cultivation was subdued to decimal dilution and poured on LB agar plates. The rest of the evaluation was carried out by the same procedure as described for CFU/ml counting of biofilm cells.

The metabolic activity of cells dispersed from the biofilm was evaluated similarly as above with slight modification according to Paldrychová et al. (2020). Briefly, the suspension was centrifuged in Eppendorf Mini Spin (Eppendorf, Czechoslovakia) for 10 min at 10,000g. The supernatant was poured away and 340 μl of filtered solution of MTT (1 mg/ml in PBS; Across Organics, USA) and 408 μl of D-Glucose (57.4 mg/ml in PBS; Penta, Czech Republic) was added to cell pellet. The pellet was resuspended, and cells were incubated at 37 °C at 150 rpm for 2 h. A formazan dissolving solution was poured to mixture in a volume of 750 μl and the incubation continued at 37 °C and 230 rpm for approximately 30 min until complete solubilization of formazan crystals. The mixture was centrifuged once again in MiniSpin for 10 min and 10,000g and the supernatant was spectrophotometrically measured at 570 nm. The experiments were carried out in three independent repetitions.

### Statistical analysis

The results were averaged, and distant values were omitted according to Dixon’s Q test. Standard deviations were calculated. The significance of results was evaluated by one-way analysis of variance (one-way ANOVA) by comparing the treatments with untreated controls with significance level of p < 0.5.

### N-acyl homoserine lactones production by biofilm affected by NTP

The N-acyl homoserine lactones (AHL) production was evaluated by biosensor assay using *Agrobacterium tumefaciens* NTL4 (pZLR4) ATCC BAA 2,240, which responds to the presence of AHL with production of β-D-galactosidase, as previously described in Paldrychová et al. (2019). AHL are extracellular substances, thus they are produced by biofilm cells together with cells dispersed from the biofilm. The cell suspension taken from the vicinity of the biofilm (the remaining liquid with cells after the coupon with biofilm was taken out of plastic container) was centrifuged for 5 min at 10,000g. The supernatant (2 µl) was mixed with 50 µl of *A. tumefaciens* cell suspension (OD_600nm_ = 0.250 ± 0.020) and cultured for 18 h at 30 °C and 150 rpm. After cultivation, 50 μl of lysis buffer was added to the suspension, which was then incubated for 90 min at 25 °C and 150 rpm to release β-d-galactosidase. Lastly, an X-gal solution (50 μl) was added to the mixture, and the cells were incubated for another 1 h at 25 °C and 150 rpm. A 100 µl aliquot from each well was measured spectrophotometrically at 660 nm. The experiments were carried out in three independent repetitions.

### LasB-elastase activity of biofilm affected by NTP

LasB-elastase activity caused by extracellular enzyme produced by biofilm cells together with cells dispersed from the biofilm was evaluated using colorimetric assay based on the digestion of Elastin-Congo Red conjugate according to Ziuzina et al. (2015). The cell suspension taken from the vicinity of the biofilm was centrifuged for 5 min at 10,000g and a volume of 700 µl of supernatant was mixed with 250 µl of Elastin-Congo Red (Sigma Aldrich, USA) dissolved in Tris-HCl buffer (pH 8, 5 mg/ml; PanReac AppliChem, Germany) in microtubes. The mixture was incubated for 24 h at 37 °C and 150 rpm. After incubation, the samples were centrifuged and 100 µl of supernatant from each tube containing split off Congo red dye was spectrophotometrically measured at 495 nm. The experiments were carried out in three independent repetitions.

### Proteases activity of biofilm affected by NTP

The proteases activity caused by extracellular enzymes produced by biofilm cells together with cells dispersed from the biofilm was evaluated using colorimetric assay based on digestion of chromogenic azocasein according to Das et al. (2016). By the digestion, azopeptides are created which are soluble in trichloroacetic acid and can be measured spectrophotometrically. The cell suspension taken from the vicinity of the biofilm was centrifuged for 5 min at 10,000 g and the supernatant in a volume of 150 µl was mixed with 1 ml of 3 mg/ml azocasein (Sigma Aldrich, Czech Republic) dissolved in 50 mM Tris-HCl (pH 8 in demineralized water). The tubes were mixed and incubated at 37 °C and 150 rpm for 30 min. The enzymatic reaction was terminated by addition of 500 µl of 10% trichloroacetic acid. The tubes were mixed and centrifuged. The 100 µl aliquot of each supernatant was measured spectrophotometrically at 400 nm. The experiments were carried out in three independent repetitions.

### Haemolytic activity of biofilm affected by NTP

The haemolytic activity caused by extracellular enzymes produced by biofilm cells together with cells dispersed from the biofilm was evaluated using defibrinated sheep blood according to Bandeira et al. (2018) with slight modification as described in Kašparová et al. (2021). The cell suspension taken from the vicinity of the biofilm was centrifuged for 5 min at 10,000g and 1 ml of supernatant from each microtube was mixed with 100 µl of 5% sheep blood diluted (LabMediaServis, Czech Republic) in PBS. The positive control represented 1 ml of 2% SDS (Oxoid, Germany) in LB medium and negative control represented 1 ml of LB medium both mixed accordingly with sheep blood. The samples were mixed thoroughly and incubated for 90 min at 37 °C. After incubation, the samples were once again mixed and 100 µl from each tube was spectrophotometrically measured at 540 nm. The haemolytic activity was assessed by linear regression where positive control was 100% haemolytic and negative control 0% haemolytic. The experiments were carried out in three independent repetitions.

### Gelatinase activity of biofilm cells and cells dispersed from biofilm affected by NTP

The gelatinase activity caused by extracellular enzyme produced by biofilm cells or cells dispersed from the biofilm was assessed according to Marra et al. (2007). In brief, the suspension of cells (1 ml) was taken from the vicinity of the biofilm into microtubes. The biofilm-covered coupons were rinsed with saline and sonicated for 15 min in 1 ml of saline to remove biofilm cells from the surface of coupons. After that, both types of samples were injected thrice into different places into nutrient broth (Carl Roth, Germany) mixed with gelatin (Across Organics, USA) in a volume of 3×5 µl in tubes. The inoculated gelatin tubes were incubated for 72 h at 37 °C. After incubation, the tubes were put to 4 °C for 30 min. If the gelatin in tube remained liquified even after cooling down, the cells in the sample produced gelatinase and therefore the sample is gelatinase positive. If the gelatin in tube remained solid after cooling down, the cells in sample did not produce gelatinase and therefore are gelatinase negative. As negative control the experiment also contained gelatin tubes with injected saline instead of sample, which remained solid when cooled down after incubation. The experiments were carried out in triplicates and the results were qualitative

### Lipases activity of biofilm cells and cells dispersed from biofilm affected by NTP

The lipases activity caused by extracellular enzymes produced by biofilm cells or cells dispersed from biofilm were evaluated using tributyrin-supplemented nutrient agar plates according to Saising et al. (2012). In short, the suspension of cells (1 ml) was taken from the vicinity of the biofilm into microtubes. The biofilm-covered coupons were rinsed with saline and sonicated for 15 min in 1 ml of saline to remove biofilm cells from the surface of coupons. After that, both types of samples were spotted on the nutrient agar (Oxoid, Germany) plate containing 1% of tributyrin (Oxoid, Germany) and 0.1% of Tween 20 (Carl Roth, Germany) in a volume of 5 µl. The spotted agar plates were then incubated for 72 h at 37 °C. The sample producing opalescent greenish halo around formed colony was deemed to be lipases positive. The sample lacking such halo around formed colony was lipases negative. The experiments were carried out in triplicates and the results were qualitative

### Visualization of biofilm cells and cells dispersed from biofilm affected by NTP using transmission electron microscopy

The effect of NTP on the morphology of *P. aeruginosa* DBM 3181 biofilm cells as well as cells dispersed from the biofilm was visualized by transmission electron microscopy (TEM) using slightly modified procedure as described in Kočendová et al. (2019). In short, the 15 and 60-min NTP-treated biofilm was rinsed with saline, removed from Ti-6Al-4V alloy coupons by sonication for 15 min in glass container containing 1 ml of saline, and after that the biofilm cells were resuspended and collected in another microtubes. The suspension of cells dispersed from the NTP-treated biofilms was removed from the vicinity of the biofilm and collected in microtubes. Both types of prepared samples were several times centrifugated (at 14,000 g for 10 min) and the pellet was resuspended in 100 μL of saline. The biofilm cells as well as cells dispersed from the biofilm were visualized by negative staining and fixed for preparation of ultra-thin sections. Briefly, parlodion carbon-coated grids were floated on the top of 10 µl drop of each sample for 5 min. The grids were washed twice on a drop of water and negative stained with 0.25% phosphotungstic acid (pH 7.4). The rest of samples were fixed for 24 hours at 4 °C in 3% glutaraldehyde in 0.1 M cacodylate buffer (pH 7.4), and thereafter for 1 hour at 4 °C in 1% osmium tetroxide. Subsequently, the samples were dehydrated by an increasing gradient of ethanol and embedded in Agar 100 resin. The ultra-thin sections (70 nm) were stained by saturated uranyl acetate solution in water and Reynold´s lead citrate depicted the entire cells. All samples were observed with a JEOL JEM 1011 operating at 80 kV. The figures are shown with a scale bar 1 µl for negative staining and 500 nm for ultra-thin sections.

### Pyocyanin production by biofilm cells and cells dispersed from biofilm affected by NTP

The production of extracellular pigment pyocyanin was assessed qualitatively using King A agar as described in Kašparová et al. (2022). In short, the suspension of cells (1 ml) was taken from the vicinity of the biofilm into microtubes. The biofilm-covered coupons were rinsed with saline and sonicated for 15 min in 1 ml of saline to remove biofilm cells from the surface of coupons. After that, both types of samples were spotted in a volume of 100 µl onto King A agar and cultured for 24 h at 37 °C. The production of pyocyanin was determined as green to blue halo around grown colonies.

The pyocyanin production was also quantified by extraction in chloroform according to Ziuzina et al. (2015). The assay was used only for the strains *P. aeruginosa* ATCC 10145, which production of pyocyanin was the most notable. The cell suspension taken from the vicinity of the biofilm was centrifuged for 5 min at 10,000 g and the supernatant in a volume of 2.5 ml was extracted with 2 ml of chloroform (Penta, Czech Republic) by shaking in a separating funnel. The chloroform bottom phase was then mixed with 1 ml of 1 M HCl (Penta, Czech Republic) and shaken properly. The phases were let to separate and upper HCl phase was poured to polymethylmethacrylate cuvette and measured spectrophotometrically at 520 nm. The experiment was carried out in triplicates.

## Results

### Inhibitory effect of NTP on biofilm cells, cells dispersed from biofilm and AHL relative level

**Figure 1:**
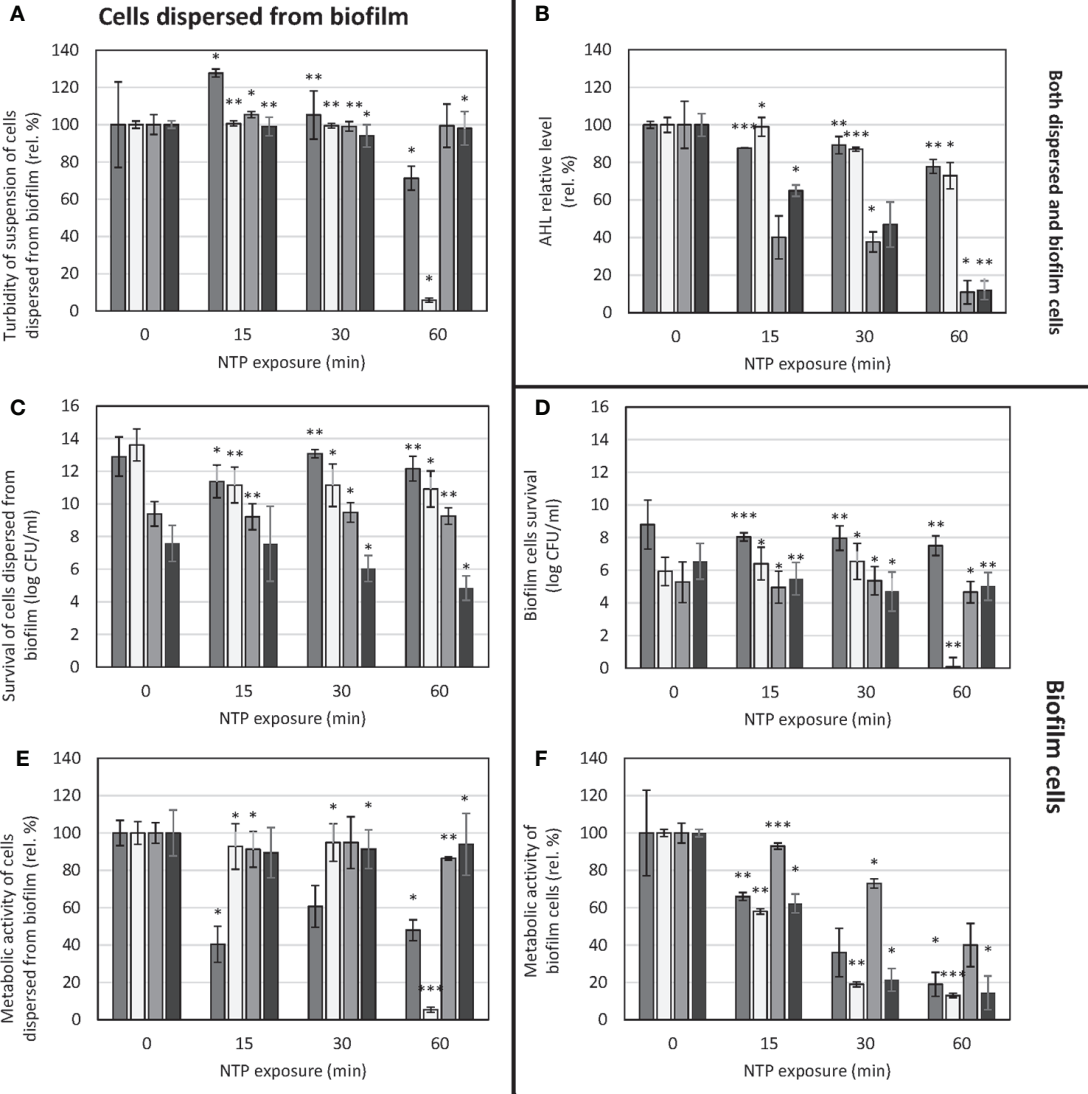
Effect of NTP exposure on survival and viability of cells dispersed from biofilm as well as biofilm cells of *Pseudomonas aeruginosa* and AHL relative level. **(A)** – turbidity of suspension of cells dispersed from biofilm, **(B)** – AHL relative level, **(C)** – survival of suspension of cells dispersed from biofilm, **(D)** – biofilm cells survival, **(E)** – metabolic activity of suspension of cells dispersed from biofilm, **(F)** – metabolic activity of biofilm cells. ■ *P. aeruginosa* ATCC 10145; 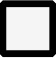
*P. aeruginosa* ATCC 15442; ■ *P. aeruginosa* DBM 3081; ■ *P. aeruginosa* DBM 3181; ANOVA: *p = 0.5-0.01, **p = 0.01-0.001, ***p< 0.001.

The assessment of the overall antibiofilm activity of NTP on *P. aeruginosa* biofilm (including biofilm cells as well as cells dispersed from the biofilm) is depicted in **Figure 1**.

The turbidity of suspension of cells dispersed from the biofilm was mostly unaffected only except for the strain *P. aeruginosa* ATCC 15442. In this case, the turbidity of cells’ suspension decreased by 94% when treated for 60 min (**Figure 1A**). Concerning survival of cells dispersed from the biofilm, in the case of *P. aeruginosa* ATCC 10145 and *P. aeruginosa* DBM 3081, the NTP treatment caused no significant decrease in CFU/ml counts (**Figure 1C**). In the case of *P. aeruginosa* ATCC 15442, although there was significant decrease in suspension turbidity as mentioned above, the survival counts dropped only from 13.6 log CFU/ml to approx. 11 log CFU/ml with each length of treatment with no further decreasing tendency. The most apparent decreasing trend was found for clinical isolate *P. aeruginosa* DBM 3181, with the greatest decrease by 3 log CFU/ml. The evaluation of metabolic activity of cells’ suspension showed the same decrease in the case of *P. aeruginosa* ATCC 15442 as seen in turbidity assay: 60-min treatment with NTP resulted in 95% decrease of viability of its cells dispersed from the biofilm (**Figure 1E**). On the contrary, the metabolic activity of clinical isolate *P. aeruginosa* DBM 3181 was not affected.

The biofilm cells’ survival was affected by NTP only slightly as CFU/ml counts in most strains decreased only by 1.5 log at maximum (**Figure 1D**). The only exception was the strain *P. aeruginosa* ATCC 15442, which survival was completely suppressed by 60-min treatment with NTP. In the case of metabolic activity of biofilm cells, the effect of NTP was much more resolute as shown in **Figure 1F**. In most cases, 60-min treatment with NTP caused at least 80% decrease in biofilm cells metabolic activity apart from *P. aeruginosa* DBM 3081 (decrease by 60%). The great decrease (by approx. 80%) was also achieved by 30-min exposure to NTP in the case of *P. aeruginosa* ATCC 15442 and clinical isolate *P. aeruginosa* DBM 3181.

In terms of AHL relative level investigation, there was noticeable decrease in most strains (**Figure 1B**). The strains *P. aeruginosa* DBM 3081 and *P. aeruginosa* DBM 3181 were the most susceptible to anti-QS effect of NTP, as the AHL relative level decreased already after 15-min treatment by 60 and 35%, respectively. The longest exposure of these two strains to NTP (60 min) resulted in almost 90% decrease of AHL relative level. Both ATCC strains were mildly susceptible, as the 60-min treatment with NTP resulted in at least 20% decline in AHL relative level.

### Effect of NTP on disruption of biofilm cells

**Figure 2:**
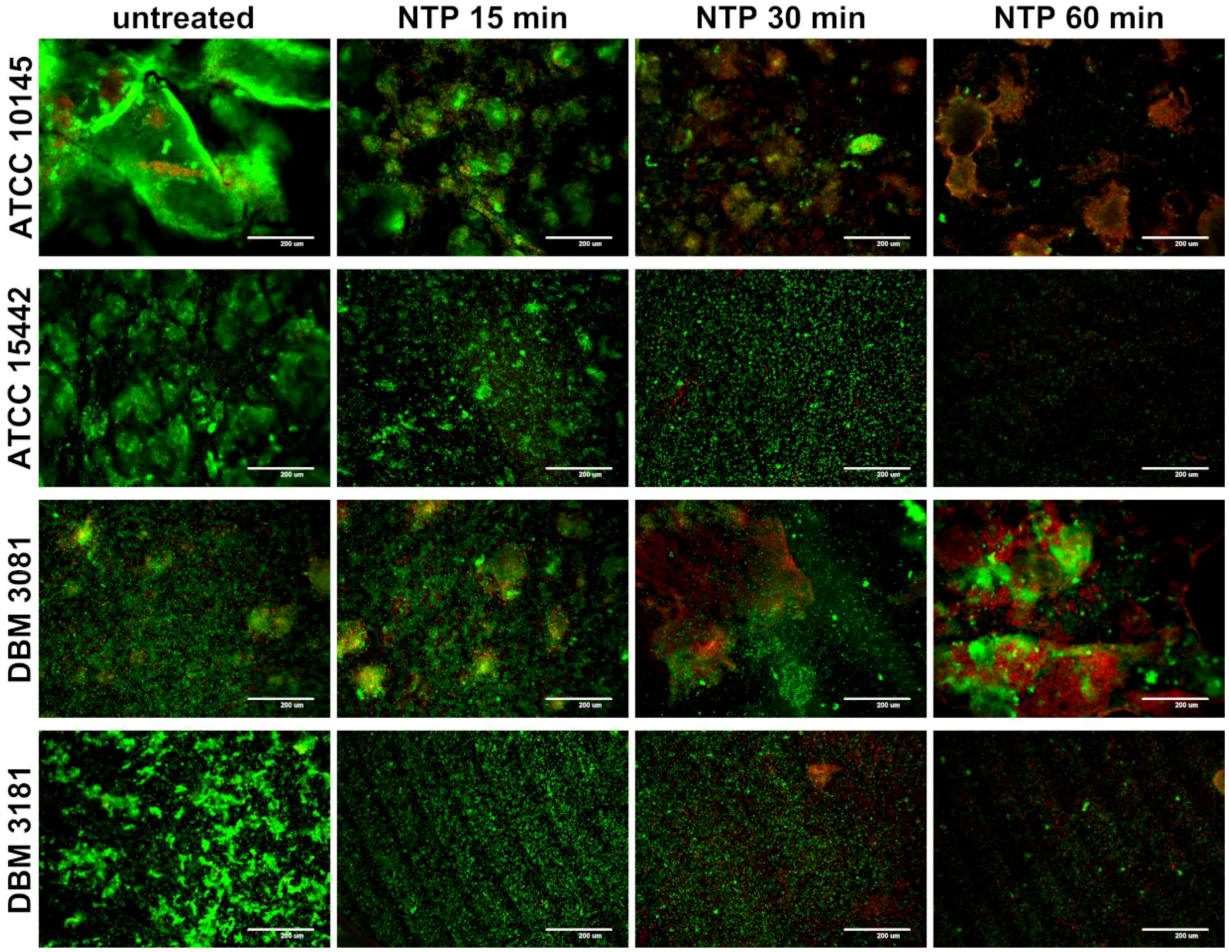
Effect of non-thermal plasma (NTP) on biofilm cells of four *Pseudomonas aeruginosa* strains visualized by spinning disc confocal microscope (SDCM) using SYTO13/propidium iodide (PI) overlay. Biofilm of *P. aeruginosa* ATCC 10145, ATCC 15442, DBM 3081 and DBM 3181 was formed on Ti-6Al-4V alloy coupons; green signal: all present cells (SYTO13); red signal: dead/disrupted cells (PI); scale bar = 200 µm.

The effect of NTP on biofilm cells of *P. aeruginosa* was visualized by SDCM using LIVE/DEAD staining as shown in **Figure 2**. All strains tested showed different architecture of biofilm formed without any treatment. The strain *P. aeruginosa* ATCC 10145 exerted the greatest ability to form very thick compact biofilm with notable extracellular matrix. The other strains formed biofilm consisting of microcolonies of cells covering the entire surface of Ti-6Al-4V alloy. The biofilm-covered area on Ti-6Al-4V coupons was decreased by NTP treatment for 15 min in all cases except *P. aeruginosa* DBM 3081 which biofilm structure remained unaffected. Despite, this strain showed small amount of disrupted or dead cells as indicated by red signals. The treatment of biofilm with NTP for 30 min resulted in greater extent of cell disruption and possible killing as the red signal became more prominent in the overlay images especially in the case of *P. aeruginosa* ATCC 10145 and DBM 3081. The longest NTP treatment (60 min) eradicated almost all biofilm cells of *P. aeruginosa* ATCC 15442 and DBM 3181 and caused significant decrease of biofilm-covered area and cell disruption and possible killing in the two remaining strains. The difference between untreated biofilm of all strains tested and biofilm treated with NTP for 60 min is even more apparent in **Figure S1** which depicts SYTO13 and PI staining in two divided frames instead of overlay.

### Effect of NTP on LasB-elastase, proteases and haemolysins production by biofilm cells together with cells dispersed from biofilm

**Figure 3:**
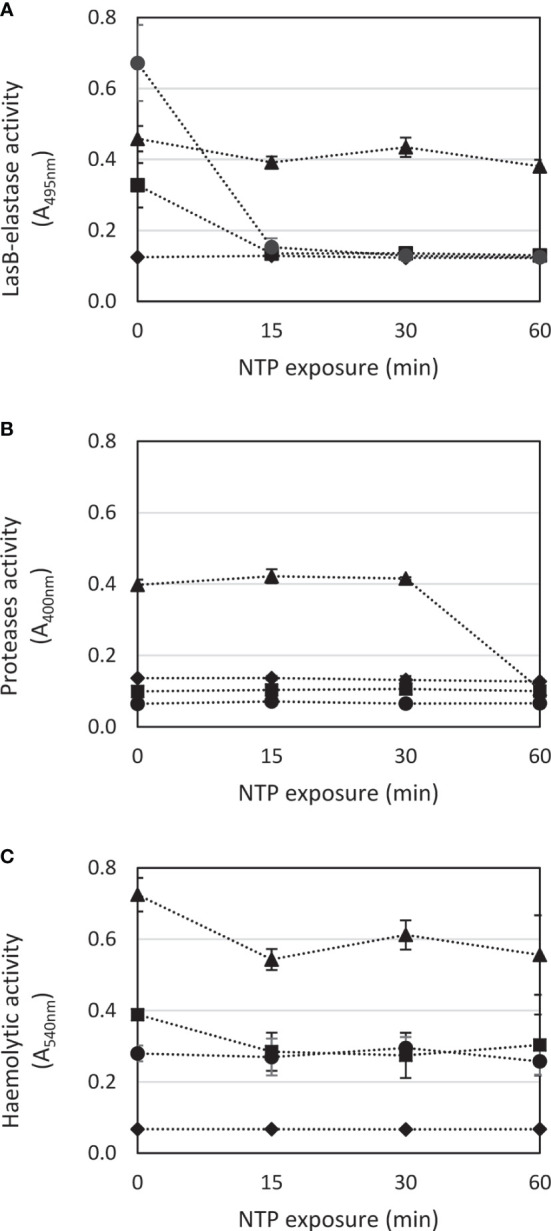
Effect of NTP exposure on virulence factors production of *Pseudomonas aeruginosa*. **(A)** – LasB-elastase activity, **(B)** – proteases activity, **(C)** – haemolytic activity. ■ *P. aeruginosa* ATCC 10145; ▲ *P. aeruginosa* ATCC 15442; ♦ *P. aeruginosa* DBM 3081; ⚫ *P. aeruginosa* DBM 3181.

The effect of NTP treatment on LasB-elastase, proteases and haemolysins production by *P. aeruginosa* (evaluated from the cell suspension taken from the vicinity of the biofilm) is depicted in **Figure 3** (the values of absorbance transferred to relative percentages, or percentages in the case haemolytic activity are shown in **Table S1**). The differences in the virulence factors production of untreated cells is shown in **Table S3**. There are apparent differences in the production of all mentioned factors by studied strains unaffected by NTP. In the case of LasB-elastase, the most virulent strain is the clinical isolate *P. aeruginosa* DBM 3181, and the least is the phenotypically atypical strain *P. aeruginosa* DBM 3081. The production of proteases was detected only by *P. aeruginosa* ATCC 15442. The potent haemolytic activity was found in most cases except for *P. aeruginosa* DBM 3081. Regarding the antivirulence effect of NTP, as shown in **Figure 3A**, there was a very large decrease of LasB-elastase activity already by treatment for 15 min in the case of the strains *P. aeruginosa* ATCC 10145 and *P. aeruginosa* DBM 3181 (by 60 and 82%, respectively; Tab. S1). On the contrary, the LasB-elastase production was almost unaffected in the strains *P. aeruginosa* ATCC 15442 and *P. aeruginosa* DBM 3081 (**Figure 3A**). There was a large decrease (by 75%) of proteases activity in the strain *P. aeruginosa* ATCC 15442 due to NTP treatment for 60 min (**Figures 3B**; **S1**). As the other strains showed almost no protease activity, of course there was no effect of NTP. Similarly, the haemolytic activity of studied strains was mostly unaffected by NTP treatment. In the most haemolytic strain *P. aeruginosa* ATCC 15442, 60-min treatment with NTP resulted in only 25% decrease in this activity (**Figure 3C**; **Table S1**).

### Effect of NTP on cell morphology of biofilm cells and cells dispersed from biofilm

**Figure 4:**
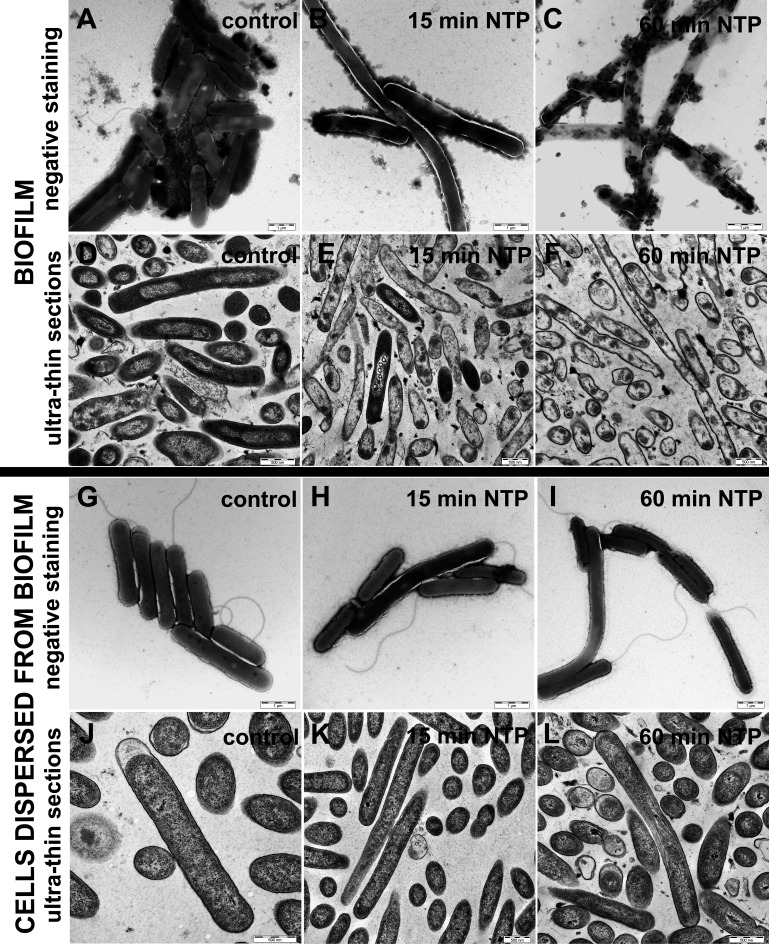
Effect of NTP on cell morphology of biofilm cells and cells dispersed from biofilm of *Pseudomonas aeruginosa* DBM 3181 visualized by transmission electron microscopy. **(A)**– biofilm untreated cells (negative staining), **(B)** – biofilm cells treated with 15 min of NTP (negative staining), **(C)** – biofilm cells treated with 60 min of NTP (negative staining), **(D)** – biofilm untreated cells (ultra-thin sections), **(E)** – biofilm cells treated with 15 min of NTP (ultra-thin sections), **(F)** – biofilm cells treated with 60 min of NTP (ultra-thin sections), **(G)** - cells dispersed from untreated biofilm (negative staining), **(H)** - cells dispersed from biofilm treated with 15 min of NTP (negative staining), **(I)** - cells dispersed from biofilm treated with 60 min of NTP (negative staining), **(J)** - cells dispersed from untreated biofilm (ultra-thin sections), **(K)** - cells dispersed from biofilm treated with 15 min of NTP (ultra-thin sections), **(L)** - cells dispersed from biofilm treated with 60 min of NTP (ultra-thin sections). Scale bars for negative staining and ultra-thin sections are 1 µm and 500 nm, respectively.

The visualization of cell morphology of *P. aeruginosa* DBM 3181 after NTP treatment was prepared for biofilm cells and cells dispersed from biofilm using TEM (**Figure 4**). The samples were prepared as negative staining (**Figures 4A–C, G–I**) or ultra-thin sections (**Figures 4D–F, J–L**).

The untreated biofilms cells were arranged in tightly connected clusters containing extracellular matrix (**Figure 4A**). Ultra-thin sections of the untreated biofilms cells depicted undamaged cells visible in longitudinal (rod shape) and transverse (circular shape) section (**Figure 4D**). On the contrary, negative stained biofilm cells treated with 15 min of NTP (**Figure 4B**) shown elongated shape or better stained cells indicating cell wall damage. In the case of ultra-thin sections, 15-min NTP treated biofilm cells had disrupted cell envelopes or disorganized cell compartments (**Figure 4E**). Finally, the treatment of biofilm cells with NTP for 60 min caused complete lysis of cells as shown in **Figure 4C**. These cells had completely disrupted cell envelopes with the cell contents poured out (**Figure 4F**).

In contrast, the visualization of cells dispersed from the biofilm of *P. aeruginosa* DBM 3181 untreated (**Figures 4G, J**) or treated with 15 or 60 min of NTP (**Figures 4H, K** or **Figures 4I, L**) clearly indicated the ability of cells dispersed from the biofilm to revitalize in an environment containing sufficient nutrients, even though the cells bound in the biofilm were severely damaged. The cells dispersed from the biofilm were undamaged in both NTP exposure times. In some cases, the shape of the cells was prolonged, but in no case the damage of cell wall or the internal contents of cells was observed.

### Effect of NTP on pyocyanine, gelatinase and lipases production by biofilm cells and cells dispersed from biofilm

**Figure 5:**
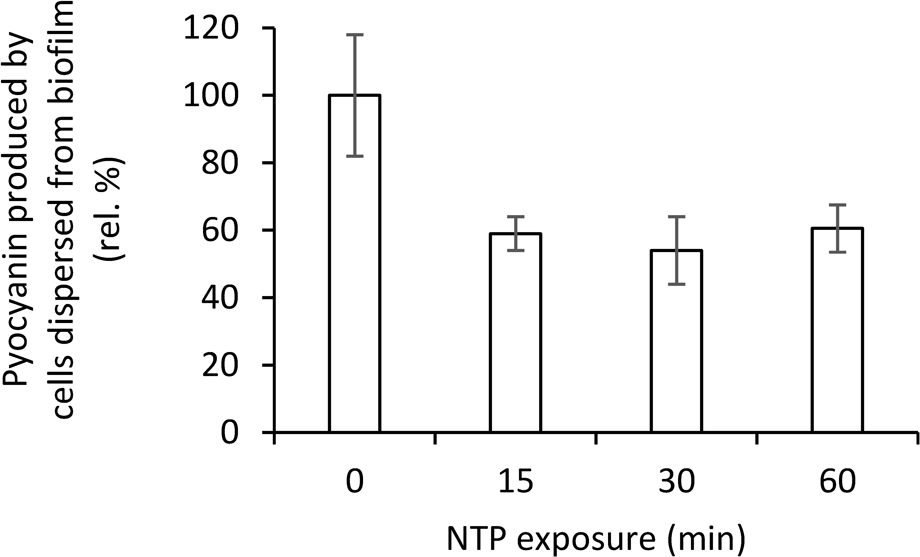
Effect of NTP treatment on pyocyanin production by cells dispersed from biofilm of *Pseudomonas aeruginosa* ATCC 10145 determined by pyocyanin extraction.

The effect of NTP treatment on pyocyanine production by biofilm cells or cells dispersed from the biofilm of four strains of *P. aeruginosa* is shown in **Table 1**. The ability to produce pyocyanin was found by most strains with the exception of *P. aeruginosa* ATCC 15442, which was completely pyocyanin negative. The highest pyocyanine production capacity was determined at *P. aeruginosa* 10145. The NTP exposure decreased pyocyanin production in all pyocyanin positive strains. In the case of *P. aeruginosa* ATCC 10145, the 60-min NTP treatment only lowered pyocyanin production by cells dispersed from the biofilm but it completely suppressed the pyocyanin production by biofilm cells. These results were also confirmed using pyocyanin extraction in this strain. The NTP treatment of *P. aeruginosa* ATCC 10145 cells dispersed from biofilm for 15-60 min caused approximately 40% decrease in pyocyanin production (**Figure 5**).

**Table 1:** Effect of NTP on pyocyanin production by biofilm cells and cells dispersed from biofilm of *Pseudomonas aeruginosa* determined by cultivation on King A agar plates.

	Pyocyanin production by biofilm cells					Pyocyanin production by cells dispersed from biofilm			
	NTP exposure (min)						NTP exposure (min)		
**Strain**	0	15	30	60	0	15	30	60
ATCC 10145	++	++	+	–	++	++	+	+
ATCC 15442	–	–	–	–	–	–	–	–
DBM 3081	+	–	–	–	+	+	+	–
DBM 3181	+	–	–	–	+	+	+	+

++, great production; +, moderate production; –, no production.

Though all strains of *P. aeruginosa* tested produced gelatinase and lipases at a similar level, there was no effect of NTP on these activities of *P. aeruginosa* strains as all samples were still positive in production even after the 60-min of NTP treatment (**Supplementary Table S2**).

## Discussion

*P. aeruginosa* belongs to the most common pathogens of today medicine, on which the effectivity of cold atmospheric or otherwise called non-thermal plasma is studied. With a large array of various technical arrangement and power regimens, the generalization of NTP effect on this species is quite difficult. In a comprehensive review worked out by our research group, we have attempted to summarize the efficacy of NTP towards pathogens of ESKAPE group (including also *P. aeruginosa*) as hinted above (Scholtz et al., 2021). The presented work focused on the antivirulence activity of cometary type of discharge for generation of NTP. Our research group developed it couple decades ago and since then its activity towards many pathogenic microorganisms including *P. aeruginosa* was tested (Scholtz and Julák, 2010a; Scholtz and Julák, 2010b; Scholtz et al., 2010; Julák et al., 2011; Julák et al., 2012; Julák et al., 2013; Scholtz et al., 2013; Scholtz et al., 2015; Khun et al., 2018; Paldrychová et al., 2019; Vaňková et al., 2019; Paldrychová et al., 2020; Vaňková et al., 2020b; Lokajová et al., 2021; Scholtz et al., 2021).

In general, the NTP treatment of biofilms usually takes from several minutes up to an hour to achieve complete inhibition of biofilm cells viability or even total reduction of survival counts (usually with untreated samples of 6-8 log CFU/ml) (Alkawareek et al., 2012; Matthes et al., 2013; Mai-Prochnow et al., 2015; Gabriel et al., 2016). Even in the case of very efficient plasma-jet arrangement, the treatment takes couple minutes as opposed to the treatment of cells in suspension, indicating increased tolerance of biofilm formed on different surfaces (Flynn et al., 2015). The biofilm formation increases endurance of cells in the lower layers towards any antimicrobial treatment up to 1,000-fold, which is believed to happen due to 70% change in phenotype, shielding by produced extracellular matrix and slower metabolism of cells in the lower layers (Costerton et al., 1999; Mah and O'Toole, 2001). An agent, which would have significant antibiofilm activity would increase susceptibility of biofilm cells towards otherwise often ineffective antibiotic treatment, as was shown for example in our previous study (Paldrychová et al., 2020).

In the presented study, we observed the ability of NTP to eliminate mature biofilm as one of the very important virulence factors towards four *P. aeruginosa* strains. However, we not only monitored the ability of NTP-treated biofilm to re-form into a vital form, but also the viability of cells dispersed from the biofilm into the nutrient-rich environment, because these cells can initiate a new cycle of biofilm formation. It was clearly demonstrated using SDCM that *P. aeruginosa* biofilm exposed to NTP was either significantly affected or even eradicated depending on the strain tested. Interestingly, the survival and metabolic activity of cells dispersed from the biofilm was almost not affected. This is in accordance with visualization by SDCM, A significant decrease in colonized area after NTP treatment was observed in all strains, which indicated possible detachment of cells. Our hypothesis is that the cells which disperse from the biofilm can revive even though the biofilm is significantly damaged, and the escaped dispersed cells continue living in planktonic form. This was supported also by TEM, as the biofilm cells were significantly disrupted, but the cells dispersed from the biofilm to planktonic form remained vital and undamaged.

Although the planktonic cells remain viable, they can be further treated by conventional antimicrobial therapy in such state. Such treatment design has been already studied in our previous work (Paldrychová et al., 2020). We used there NTP to facilitate the eradication of the mature biofilms of *P. aeruginosa* on Ti-6Al-4V coupons by the subsequent addition of the solution of antibiotics (up to a 70% increase in the antibiofilm activity of gentamicin in combination with 30 min NTP treatment was achieved). Similar enhancement of antibiotic action was also studied by Gupta et al. (2017), who treated *P. aeruginosa* biofilms on titanium coupons with chlorhexidine and NTP or by Helgadóttir et al. (2017), who studied pre-treatment of *P. aeruginosa* with vitamin C to enhance subsequent treatment with NTP (Helgadottir et al., 2017).

Therefore, a beneficial effect of NTP treatment in combined therapy with other antimicrobial agents is evident. On the contrary, in addition to the apparent inhibitory effect of NTP on *P. aeruginosa* biofilm cells, current knowledge about the effect of NTP on other *P. aeruginosa* virulence factors is very limited. There are only a few studies from recent years reporting the effect of various NTP sources on virulence factors like pyocyanin, pyoverdine, proteases or LasB-elastase production (Barczak and Hung, 2009; Baron, 2010; Flynn et al., 2016; Maura et al., 2016; Barakat et al., 2019; Wang et al., 2020). Barakat et al. (2019) studied several virulence factors of *P. aeruginosa* like endotoxin production or lipopolysaccharides content when treated with NTP generated by plasma jet Barakat et al. (2019). Endotoxin concentration decreased by more than a half after already 2 min NTP treatment and diminished completely after 8 min treatment. They also explored how treatment with NTP affects content of lipopolysaccharides in cell membrane of *P. aeruginosa*, which were then extracted, and 20 g/l were administered to *Galleria mallonella* larvae. The group of larvae with lipopolysaccharides extracted from NTP-untreated *P. aeruginosa* died faster.The treatment of *P. aeruginosa* with NTP resulted in decreased negative effect of the extracted lipopolysaccharides on *G. mallonella* thus expressing antivirulence activity of NTP *in vivo*. Wang et al. (2020) used plasma jet based device for generation of NTP on suspension cells of *P. aeruginosa* and observed the production of virulence factors pyocyanin, elastase and proteases similarly to our study, but only in a planktonic form which is strikingly different from biofilm environment and can produce completely different results. The significant reduction by more than 85% was achieved for all mentioned virulence factors with treatment for 10 min. Nevertheless, it must be noted, that such similar decrease was apparent also in the determination of CFU/ml and viability using metabolic assay, thus such decrease in virulence factors production might have been caused only by correlating decrease in the number of viable cells. Antivirulence activity is significant only in the case of not affecting viability of treated cells as opposed to decrease in virulence factors production (Barczak and Hung, 2009; Baron, 2010; Maura et al., 2016). In our study, we focused on several virulence factors, specifically biofilm re-development, LasB-elastase, proteases, haemolytic, lipases and gelatinase activity and pyocyanin production. As the studied strains were purposely heterogenous, their ability to produce distinct virulence factors was also variable. Based on this diversity, antivirulence effectivity of NTP generated by used cometary discharge also differed. Las-B elastase activity was decreased already by 15-min treatment with NTP in two studied strains (type strain ATCC 10145 and clinical isolate DBM 3181), whereas it was unaffected in *P. aeruginosa* ATCC 15442. Nevertheless, this strain had significantly decreased proteases activity by NTP after 60 min treatment and although in general, there was no significant antihaemolytic activity of NTP, in this strain the haemolytic activity decreased by 20-30% by 15-60 min NTP-treatment. Concerning production of pyocyanin, an important previously mentioned pigment, NTP treatment for 15 min suppressed its production by biofilm cells completely in two strains (atypical strain DBM 3081 and clinical isolate DBM 3181) and after 60 min also in type strain ATCC 10145 (*P. aeruginosa* ATCC 15442 is pyocyanin-negative). However, the cells dispersed from the biofilm maintained their ability to produce this pigment and even 60-min NTP-treatment was not sufficient to abolish its production in the strain DBM 3181 and ATCC 10145. In the case of *P. aeruginosa* ATCC 10145, such findings were further verified also by extraction of pyocyanin from cells dispersed from the biofilm, which showed 40% decrease by all NTP exposure times equally. These findings show interesting antivirulence properties of NTP on some virulence factors such as extracellular hydrolases or pigments, but it should be borne in mind that this activity is completely strain-dependent and thus none of the virulence factors (other than the suppression of biofilm re-development) can be identified as a key factor influencing by NTP exposure. At the same time, it is not possible to say that NTP affects all virulence factors since gelatinase or lipases activity was not affected by NTP treatment at all, though.

Interestingly, the AHL relative level was decreased only in two studied strains, therefore it cannot be concluded, that such antivirulence action would be strictly anti-QS derived. To the best of our knowledge, there is only one another not yet mentioned study dealing with the effect of NTP on virulence of *P. aeruginosa*. Flynn et al. (2016) focused on the production of pyoverdine and pyocyanin when treated with plasma jet. Pyoverdine production decreased significantly already with treatment for 30 s as shown by decrease in absorbance *A*_550nm_ from approx. 450 to less than 50. Also, production of pyocyanin dropped significantly from *I*_695/550nm_ = 0.010 to less 0.0025 after treatments for 1-4 min. We also observed a decrease in pyocyanin production after 15 min, which seems to be much longer than in previously described study, but the difference corresponds to various NTP sources used. Overall, both current literature and our research indicated potential antivirulence activity of NTP towards *P. aeruginosa*. The antivirulence activity including antibiofilm action was proved in several studies to enhance other antimicrobial substances action and increase biofilm cells susceptibility. Our insight into its antivirulence action might help better understanding the mechanism of action of NTP on microorganisms and how to probably use it in medicine.

In conclusion, NTP generated by cometary discharge was proved to be an efficient agent exhibiting antivirulence effect including abolishment of re-development of *P. aeruginosa* biofilm. It decreased biofilm cells viability, suppressed some of the important virulence factors production (depending on the strain monitored), especially LasB-elastase, proteases and pyocyanin production, lowered AHL relative level and caused dispersion of the cells back into planktonic form. It was observed that although biofilm cells were rapidly killed (disrupted cell surface), their escape to planktonic form caused sustained viability despite the NTP treatment (no effect on cell morphology) as shown by TEM. The forcing of biofilm cells to switch once again to planktonic form of growth would benefit combined treatment with antibiotics which would be able to kill the planktonic cells as opposed to problematic penetration into biofilm formed on surfaces and thus inability to kill the biofilm cells sheltered inside. NTP is therefore proved to be suitable therapeutic option for biofilm-related infections by *P. aeruginosa* with low purchase cost and easy handling.

## Data availability statement

The original contributions presented in the study are included in the article/**Supplementary Material**. Further inquiries can be directed to the corresponding author.

## Author contributions

Conceptualization: MP, EV, PK. Methodology: MP, PK, RH. Formal analysis and investigation: PK, AS, MP, RH. Resources: IJK, VS, JM. Writing - Original Draft: PK and EV. Writing - Review and Editing: EV, VS, JM. Supervision: VS and JM. Funding acquisition: IJK, VS, RH. All authors contributed to the article and approved the submitted version.

## Funding

This work was supported by GACR project, grant number 22-13745S; the European Regional Development Fund; OP RDE, grant number CZ.02.1.01/0.0/0.0/16_019/0000729 and RVO project, grant number 61388963.

## Conflict of Interest

The authors declare that the research was conducted in the absence of any commercial or financial relationships that could be construed as a potential conflict of interest.

## Publisher’s note

All claims expressed in this article are solely those of the authors and do not necessarily represent those of their affiliated organizations, or those of the publisher, the editors and the reviewers. Any product that may be evaluated in this article, or claim that may be made by its manufacturer, is not guaranteed or endorsed by the publisher.
