# Supplementary material for: Non-thermal plasma causes Pseudomonas aeruginosa biofilm release to planktonic form and inhibits production of Las-B elastase, protease and pyocyanin
Source: Front Cell Infect Microbiol. 2022 Sep 23;12:993029. doi: 10.3389/fcimb.2022.993029 (PMC9544392; doi:10.3389/fcimb.2022.993029)
Supplement: Supplementary file 1 [file DataSheet_1.docx]

Supplementary materials


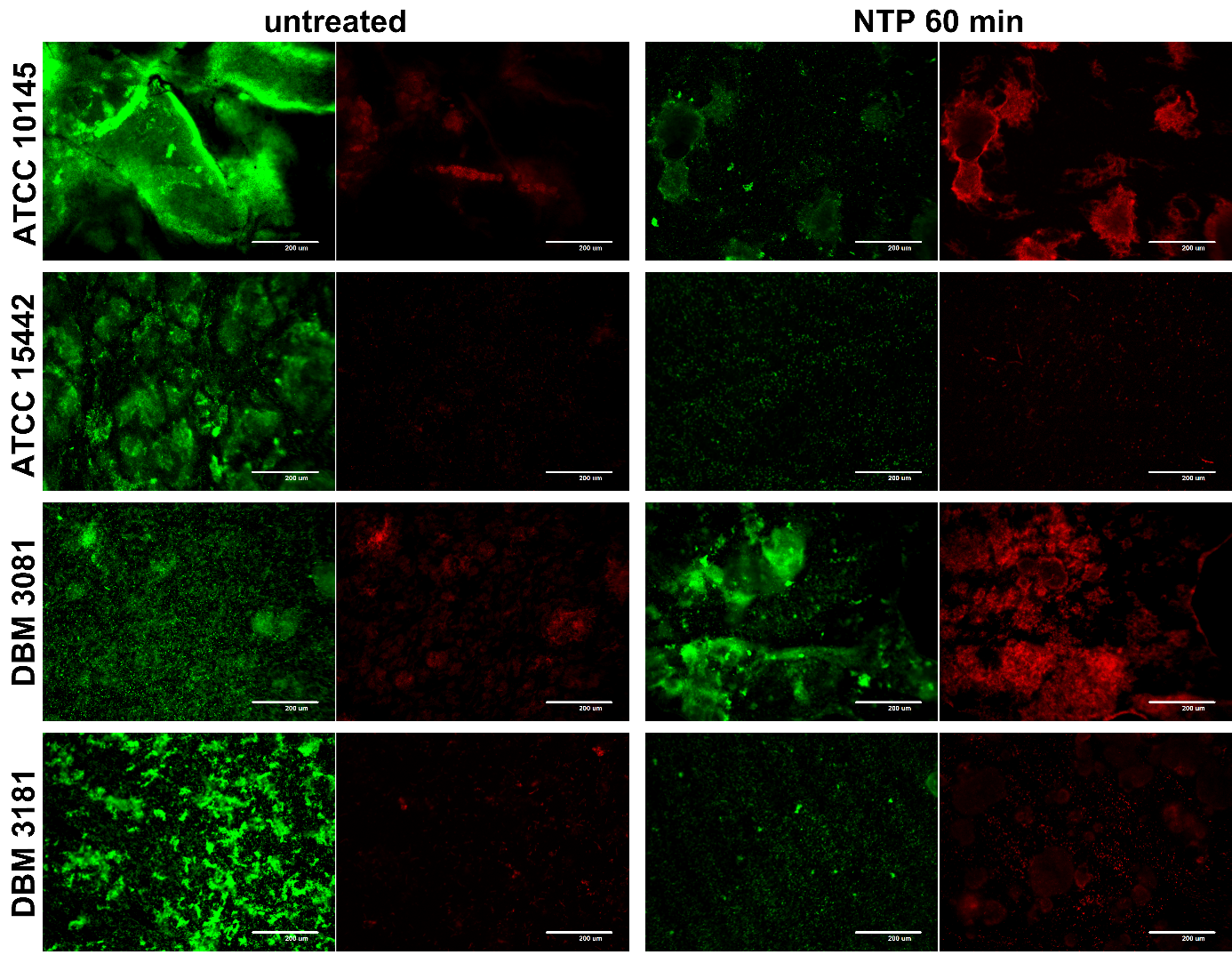


Figure S1. Comparison of untreated biofilm of four *Pseudomonas aeruginosa* strains and biofilm treated with non-thermal plasma (NTP) for 60 min visualized by spinning disc confocal microscope (SDCM) using SYTO13 and propidium iodide PI probes. Biofilm of *P. aeruginosa* ATCC 10145, ATCC 15442, DBM 3081 and DBM 3181 was formed on Ti-6Al-4V alloy coupons; green signal: all present cells (SYTO13); red signal: dead/disrupted cells (PI); scale bar = 200 µm

Table S1: Effect of NTP on virulence factor production of *Pseudomonas aeruginosa* depicted in relative percentage in the case of LasB-elastase and proteases activity and percentage calculated from positive and negative control in the case of haemolytic activity.

|  | **LasB-elastase activity (rel. %)** | | | |  | **Proteases activity (rel. %)** | | | |  | **Haemolytic activity (%)** | | | | |
| --- | --- | --- | --- | --- | --- | --- | --- | --- | --- | --- | --- | --- | --- | --- | --- |
|  | NTP exposure (min) | | | |  | NTP exposure (min) | | | |  | NTP exposure (min) | | | | |
| **Strain** | 0 | 15 | 30 | 60 |  | 0 | 15 | 30 | 60 |  | 0 | 15 | 30 | 60 |  |
| ATCC 10145 | 100 | 41 | 42 | 40 |  | 100 | 104 | 107 | 100 |  | 48 | 33 | 31 | 35 |  |
| ATCC 15442 | 100 | 85 | 95 | 83 |  | 100 | 106 | 104 | 27 |  | 96 | 70 | 80 | 72 |  |
| DBM 3081 | 100 | 103 | 99 | 98 |  | 100 | 101 | 97 | 93 |  | 1 | 1 | 1 | 1 |  |
| DBM 3181 | 100 | 23 | 19 | 19 |  | 100 | 110 | 100 | 102 |  | 32 | 30 | 34 | 29 |  |

Table S2: Qualitative determination of NTP action on lipases and gelatinase activity produced by biofilm cells and cells dispersed from biofilm affected by NTP four strains of *Pseudomonas aeruginosa*.

|  | **Lipases activity (+/˗)** | | | | | | | | | |
| --- | --- | --- | --- | --- | --- | --- | --- | --- | --- | --- |
|  | Biofilm cells | | | |  |  | Cells dispersed from biofilm | | | |
|  | NTP exposure (min) | | | |  |  | NTP exposure (min) | | | |
| **Strain** | 0 | 15 | 30 | 60 |  |  | 0 | 15 | 30 | 60 |
| ATCC 10145 | + | + | + | + |  |  | + | + | + | + |
| ATCC 15442 | + | + | + | + |  |  | + | + | + | + |
| DBM 3081 | + | + | + | + |  |  | + | + | + | + |
| DBM 3181 | + | + | + | + |  |  | + | + | + | + |
|  |  | | | |  |  |  |  |  |  |
|  | **Gelatinase activity (+/˗)** | | | | | | | | | |
|  | Biofilm cells | | | |  |  | Cells dispersed from biofilm | | | |
|  | NTP exposure (min) | | | |  |  | NTP exposure (min) | | | |
| **Strain** | 0 | 15 | 30 | 60 |  |  | 0 | 15 | 30 | 60 |
| ATCC 10145 | + | + | + | + |  |  | + | + | + | + |
| ATCC 15442 | + | + | + | + |  |  | + | + | + | + |
| DBM 3081 | + | + | + | + |  |  | + | + | + | + |
| DBM 3181 | + | + | + | + |  |  | + | + | + | + |

Table S3: Overview of the extent of virulence factors production of untreated studied strains of *P. aeruginosa*

|  | **Strain** | | | | | | | |
| --- | --- | --- | --- | --- | --- | --- | --- | --- |
|  | ATCC 10145 | | ATCC 15442 | | DBM 3081 | | DBM 3181 | |
| **Virulence factor** | Dispersed cells | Biofilm cells | Dispersed cells | Biofilm cells | Dispersed cells | Biofilm cells | Dispersed cells | Biofilm cells |
| Las-B-elastase* | 0.33 | | 0.46 | | 0.12 | | 0.67 | |
| Proteases* | 0.10 | | 0.40 | | 0.14 | | 0.06 | |
| Haemolysins* | 0.39 | | 0.72 | | 0.07 | | 0.28 | |
| Gelatinase | + | + | + | + | + | + | + | + |
| Lipases | + | + | + | + | + | + | + | + |
| Pyocyanin | ++ | ++ | – | – | + | + | + | + |

*determined from both suspension and biofilm cells at once
